# Discovery of *circa* 115,000-year-old bone retouchers at Lingjing, Henan, China

**DOI:** 10.1371/journal.pone.0194318

**Published:** 2018-03-12

**Authors:** Luc Doyon, Zhanyang Li, Hao Li, Francesco d’Errico

**Affiliations:** 1 Centre National de la Recherche Scientifique, UMR 5199 –PACEA, Université de Bordeaux, Pessac CEDEX, France; 2 Départment d’anthropologie, Université de Montréal, Montréal, Québec, Canada; 3 Institute of Cultural Heritage, Shandong University, Jinan, Shandong, China; 4 Henan Provincial Institute of Cultural Relics and Archaeology, Zhenzhou, Henan, China; 5 Key Laboratory of Vertebrate Evolution and Human Origins, Institute of Vertebrate Paleontology and Paleoanthropology, Chinese Academy of Sciences, Beijing, Beijing Municipality, China; 6 CAS Center for Excellence in Life and Paleoenvironment, Beijing, Beijing Municipality, China; 7 SFF Centre for Early Sapiens Behaviour (SapienCE), University of Bergen, Bergen, Hordaland, Norway; Max Planck Institute for the Science of Human History, GERMANY

## Abstract

Most Chinese lithic industries dated between 300,000 and 40,000 are characterized by the absence of Levallois debitage, the persistence of core-and-flake knapping, the rarity of prepared cores, their reduction with direct hard hammer percussion, and the rarity of retouched flakes. Here we report the discovery of seven bone soft hammers at the early hominin Lingjing site (Xuchang County, Henan) dated to 125,000–105,000. These artefacts represent the first instance of the use of bone as raw material to modify stone tools found at an East Asian early Late Pleistocene site. Three types of soft hammers are identified. The first consists of large bone flakes resulting from butchery of large herbivores that were utilized as such for expedient stone tools retouching or resharpening. The second involved the fracture of weathered bone from medium size herbivores to obtain elongated splinters shaped by percussion into sub-rectangular artefacts. Traces observed on these objects indicate intensive and possibly recurrent utilization, which implies their curation over time. The last consists of antler, occasionally used. Lingjing bone tools complement what we know about archaic hominin cultural adaptations in East Asia and highlight behavioural consistencies that could not be inferred from other cultural proxies. This discovery provides a new dimension to the debate surrounding the existence of the Middle Palaeolithic in the region. The attribution of East Asian sites to the Middle Palaeolithic assumes that cultural traits such as the Levallois method represent evolutionary hallmarks applicable to regions of the world different from those in which they were originally found. Here, we promote an approach that consists in identifying, possibly from different categories of material culture, the original features of each regional cultural trajectory and understanding the behavioural and cognitive implications they may have had for past hominin populations.

## Introduction

A key issue for research on the cultural evolution of our genus is when, how, and why prehistoric populations ceased to consider bone as a by-product of hunting and carcass processing, and recognized its technological utility as a suitable raw material for other activities such as knapping and retouching of stone tools. The use of osseous artefacts in these tasks allows for a better determination of lithic blanks and retouched tools morphology [1]. Bone fragments used for knapping and retouching were signalled in the literature since the end of the 19^th^ century (for a review, see [2]). Henri-Martin [3] was the first archaeologist to propose that faunal remains from the Mousterian layers of La Quina, Charente, France, with surfaces bearing pits and scores were used for the manufacture of lithic artefacts. Subsequently, Semenov [4] interpreted microscopic features on bone from Eastern European Middle and Upper Palaeolithic sites, such as Kiik-Koba and Kostenki, as implements used for knapping stone tools and inferred laterality from the orientation of the marks. Since then, traces left on bone fragments by their experimental use as retouchers have been widely described in the scientific literature. Areas bearing these modifications are covered by “deep, short, sub-parallel, closely clustered grooves, V-shaped in cross section” [5]. More recent experiments sought to distinguish marks produced by retouching flint and quartzite blanks [6]. Sub-spherical pits and sinuous scores with rough internal morphology are characteristic of retouchers used on quartzite; trihedral pits and rectilinear scores with even internal morphology are typical of flint retouching. On archaeological specimens, these modifications are present in single or multiple areas, generally close to the bone ends [7,8].

Bone remains bearing modification interpreted as the result of their use as retouchers and soft hammers were reported at numerous sites from Europe, the Levant, and Africa. In Africa, the earliest instance of this behaviour is represented by a soft hammer on a giraffe astragalus found at Olduvai Gorge, Tanzania, in an occupation level dated between 2.1–1.5 Ma BP [9]. In the Levant, the first known soft hammer is an antler base from Gesher Benot Ya‘aqov dated to the MIS18 [10]. In Europe, the distal portion of a red deer femur bearing pits due to its use as hammer is found at Boxgrove and dates back to MIS13 [11].

In Africa, a long-bone shaft fragment from a Class III bovid used as a retoucher was uncovered in a layer attributed to the Still Bay (*circa* 77–75 ka BP) at Blombos Cave, South Africa [12]. In Europe, this tool type appears in the archaeological record between the MIS12 and 10 with a number of equid, bovid, and cervid long bone, rib, and vertebra fragments used as bone retouchers. These specimens were unearthed from Schöningen (MIS12) [13], Terra Amata [14,15], and Cueva del Angel (MIS11) [14]. From MIS9, bone retouchers become a common feature of Neanderthal toolkits both in Europe and in the Levant as attested at sites such as Bolomor Cave [16], Cagny l’Épinette [14], Gran Dolina [17], La Micoque [18], Orgnac 3 [14,15,19], Qesem Cave [16], and Schöningen [20]. This tool type is frequently identified in Middle and Upper Palaeolithic assemblages (e.g., [6,15,21–29]). A fragment of Neanderthal skull from the Mousterian site of La Quina, Charente, France [30], and a femur shaft fragment from Goyet, Belgium [31], were used as retouchers and represent the first instances of the use of human bones as tools. At present, it is unclear whether or not this technological innovation occurred in East Asia and, if so, when and where it appeared. The eastern-most Palaeolithic site that has yielded bone retoucher is Chagyrskaya Cave, Altai Mountains, where this tool was recently found in Mousterian levels [32] attributed to the end of MIS4 and the onset of MIS3 [33].

In this paper, we describe bone retouchers recovered at the Lingjing site (Xuchang, Henan, China) in a level dated to *circa* 125–105 ka BP. These artefacts represent the first evidence from Eastern Asia for the use of bone as raw material to modify stone tools. This discovery has implications for the ongoing debate on the nature of Late Pleistocene cultural adaptations in China. The lithic technology that characterizes most Chinese assemblages attributed to this period is interpreted either as reflecting a peculiar facies of the Middle Palaeolithic [34,35] or the persistence of essentially Lower Palaeolithic cultural traditions [36–39]. The Lingjing bone retouchers and the behavioural consistencies their analysis highlights show that in spite of the apparent simplicity of lithic reduction sequences identified at the site [40], Lingjing hominins integrated in their behavioural repertoire the use of bone fragments to shape stone tools. These results corroborate the view that early Late Pleistocene cultural adaptations from China must be understood as reflecting original cultural trajectories whose degree of complexity cannot be evaluated solely through the study of lithic assemblages.

## Archaeological context

**Fig 1:**
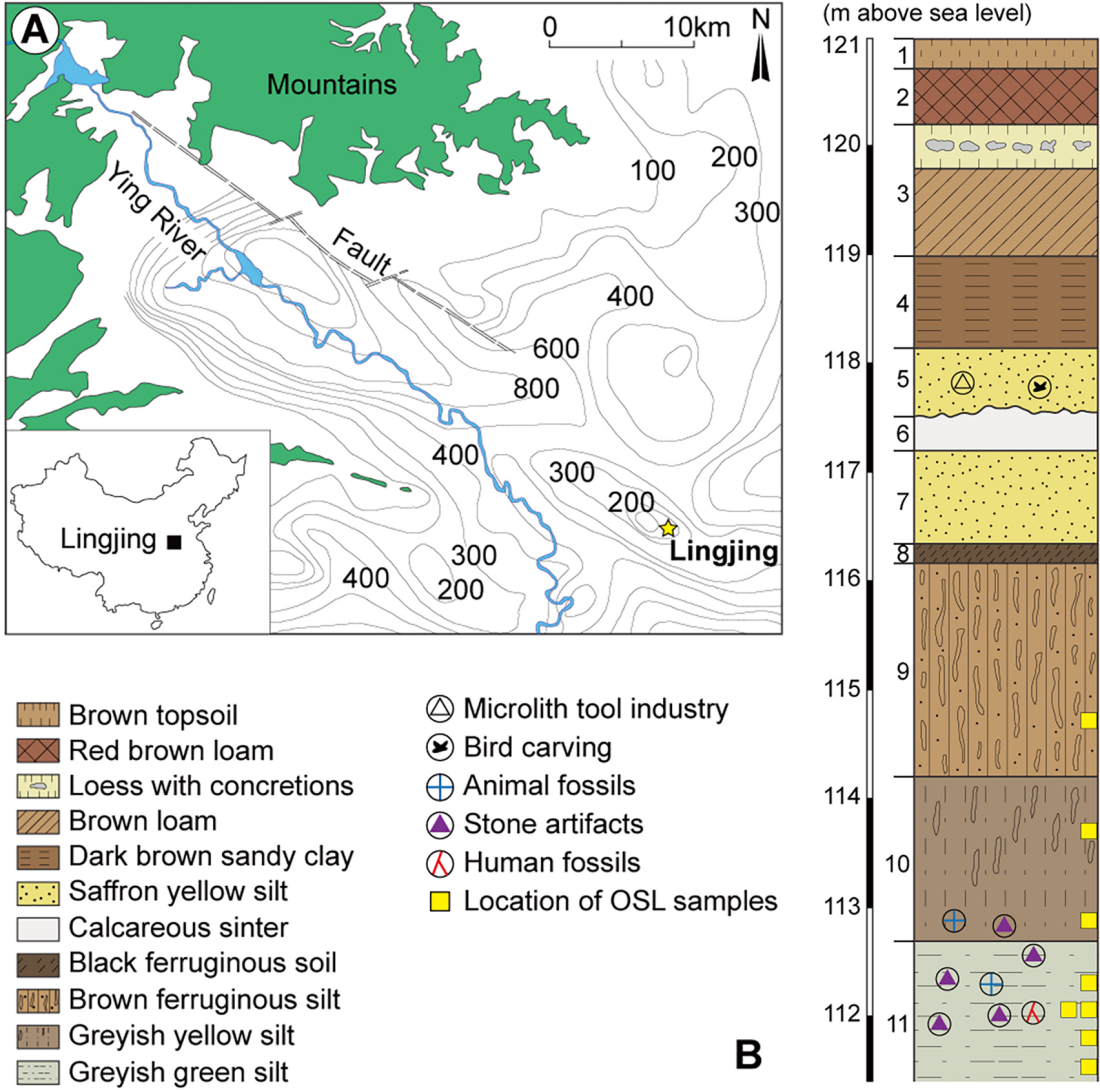
a) Location of Lingjing (Henan, China); b) Stratigraphy indicating the geological and cultural layers. Modified from [44]. Reprinted with permission from AAAS.

The Lingjing site (34° 04′ 08.6″ N, 113° 40′ 47.5″ E, elev. 117 m) is located in Lingjing town, northwest Xuchang County, Henan Province, northern China, about 120 km south of the Yellow River (Fig 1). The site was discovered in 1965 when microblades and microcores [41], as well as mammalian fossils were collected on the surface. It consists of a water-lain deposit owing to the presence of a still active water spring. The site covers an area greater than 10,000 m^2^. Since 2005, a 551-m^2^ area was systematically excavated under the supervision of one of us (LZ), and eleven geological layers were identified for a current depth of *circa* 9 meters. From the top to the bottom, layers 1–4 are Holocene in age, and have yielded material culture associated with a period that spans the Shang-Zhou Bronze Age to the Neolithic. In layer 5, microblades, microcores, bone artefacts, perforated ostrich eggshells, and ochre as well as faunal remains attest of an occupation of the site between the LGM and the Younger Dryas, a period when pottery first appears in this region [42,43]. In layer 10, a relatively small number of lithic artefacts and faunal remains were discovered. Finally, layer 11 yielded an abundant lithic and faunal assemblage, in association with two incomplete human skulls. These skulls are interpreted as bearing a mosaic of morphological features indicating both regional continuity and interregional population dynamics [44,45]. All other layers (6 to 9) are sterile.

Layer 11 was OSL dated to *circa* 125–105 ka BP by comparing the results obtained from six different OSL dating methods, including the single aliquot regenerative dose protocol [46]. This age indicates the human occupation of the site occurred during the early phases of MIS5 (MIS5e to MIS5d). Artefacts made of quartz and quartzite dominate the lithic assemblage (>99%), and include cores, flakes, formal tools (i.e., scrapers, notches, denticulates, borers, points, choppers, etc.), and debris. Chert is also marginally represented (<1%). The abundance of debitage and evidence for use wear on lithic artefacts suggests that both the manufacture and use of lithic tools occurred at the site [40].

The faunal assemblage is typical of the late Middle to early Late Pleistocene in China. The most represented species are *Equus caballus*, *Equus hemionus*, and *Bos primigenius*. They are followed by *Megaloceros ordosianus*, *Cervus elaphus*, *Coelodonta antiquitatis*, *Procapra przewalskii*, *Dicerorhinus mercki*, *Pachycrocuta* cf. *sinensis*, *Palaeoloxodon* sp., *Viverra* cf. *zibetha*, *Ursus* sp., *Sus lydekkeri*, *Hydropotes pleistocenica*, and *Axis shansius lingjingensis* subsp. nov. [47,48]. The skeletal element profile combined with the high frequency of cut marks (~34%) and their location on the bone are consistent with an interpretation of Lingjing Layer 11 as a kill-butchery site [49–52]. The proportion of bones bearing anthropogenic modifications is certainly underestimated due to the presence of thin concretions and manganese coatings on many remains, likely owing to the underground spring context in which they accumulated. The artefacts described in this study come exclusively from layer 11.

## Material and methods

The faunal assemblage from Lingjing (*N* > 50,000) is curated at the Henan Provincial Institute for Cultural Relics and Archaeology, ZhengZhou, China. The artefacts described in this study were identified during a project that aimed at reassessing the anthropogenic nature of flakes removals present on the periosteal and endosteal aspects of the faunal remains, which are interpreted as evidence of expedient osseous technologies [53,54].

A randomly selected sample of 227 bone fragments from the 2005–2015 excavations was studied with a Leica Wild M3C stereomicroscope equipped with a Nikon CoolPix 900 Digital Camera at magnifications ranging from 4x to 40x. Remains largely covered with concretions and manganese deposits were excluded from the analysis. Macroscopic photographs of the artefacts were taken with a Canon PowerShot 100 and a Nikon D300 AF Micro Nikkor 60 mm f/2.8D. Anthropogenic modifications were distinguished from natural ones on the basis of criteria known in the literature [55–58]. Identification of traces produced by the use of bone as retouchers, i.e., clusters of scores and pits present near the edge of a bone fragment, was based on experimental and archaeological studies [6,9,12,14,16,21,22,25–29,59–63]. Morphometric data were collected using a digital calliper and included the maximum length, width, and thickness of the bone fragment used as retoucher. When identifiable, the species and anatomical elements selected were recorded. Otherwise, an animal size class was estimated from the cortical thickness of the fragment. The number of pits, scores, and scale removals were counted. Traces of use as retoucher are described in accordance to the terminology proposed by Mallye *et al*. [6]. When grouped in clusters, the maximum length and width of the concentration of marks were also recorded, as well as the orientation of the cluster relative to the tool’s main axis [61–63].

## Results

### Bone modifications

Root etching is the main non-human post-depositional alteration recorded on the Lingjing faunal assemblage (22.03%; Table 1). Surfaces not affected by this process are exceptionally well preserved and allow for a precise identification of anthropogenic modifications. Carnivore gnawing and etching due to digestion were recorded on respectively 3.96% and 2.20% of the sample. Hyena remains are signalled in the faunal spectrum [47] and hyena coprolites were found [64,65]. The low percentage of faunal remains modified by carnivores, the high cranial-postcranial ratio, the high percentage of cut-marked bones (see below), the presence of complete epiphysis, and the mortality profile dominated by prime adults [51] suggest that hyenas played a marginal role in the accumulation of the bone assemblage, and only had a secondary access to carcasses [66].

**Table 1:** Bone modifications recorded on Lingjing faunal remains analysed in this study.

	*n*	Non-human			Human			
		Root Etching	Carnivore	Digestion	Cutmarks	Marrow Extraction	Retouchers	Burnt Bone
**Total**	**227**	**50**	**9**	**5**	**54**	**8**	**8**	**3**
**Percentage**	**100%**	**22.03%**	**3.96%**	**2.20%**	**23.79%**	**3.52%**	**3.52%**	**1.32%**

Cut marks are the main anthropogenic modification recorded on the faunal assemblage (23.79%). Other modifications include percussion marks, possibly for marrow extraction (3.52%), traces of utilization as retouchers (3.52%), and staining produced by heat (1.32%).

### Bone retouchers

**Fig 2:**
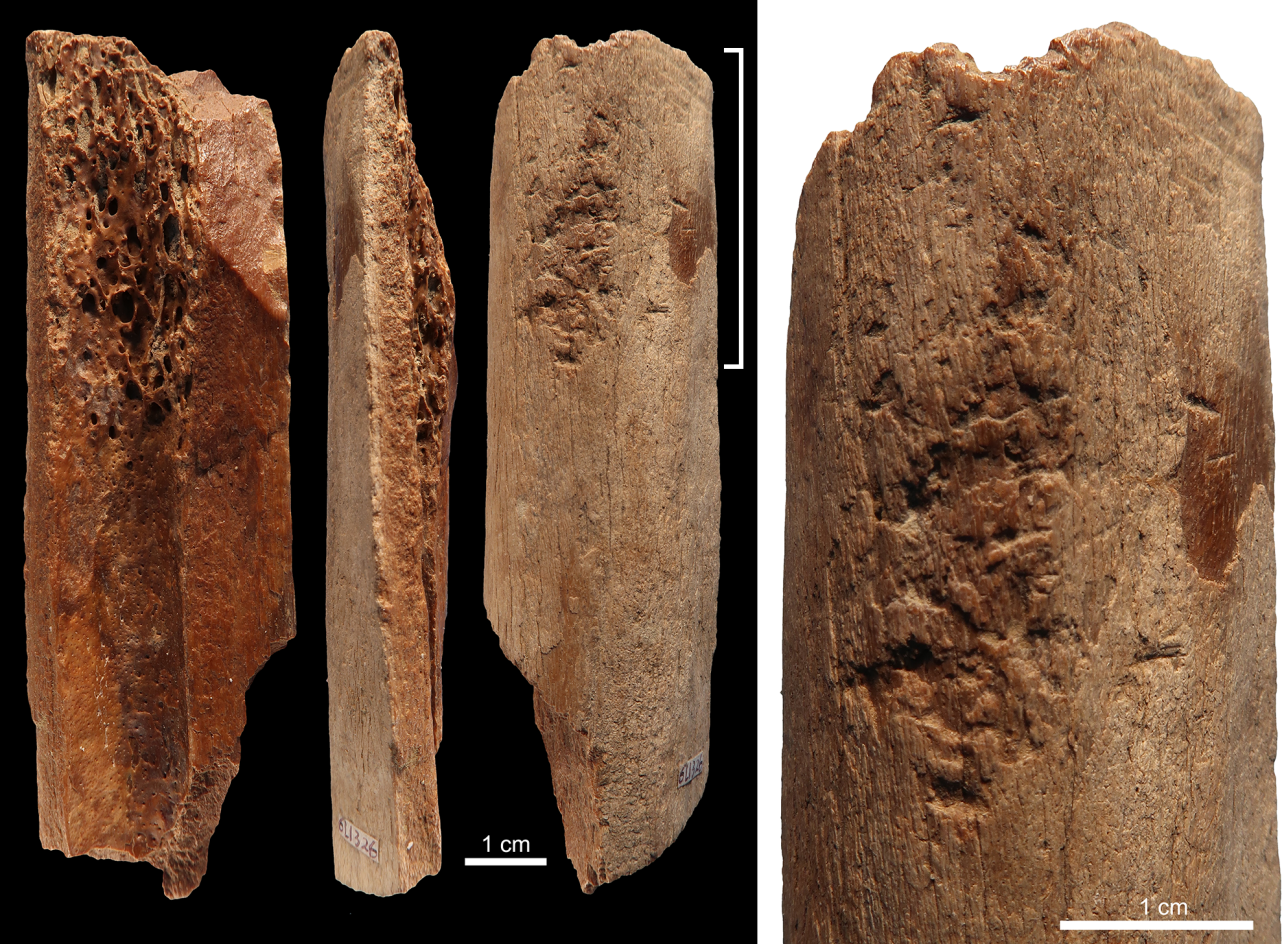
Retoucher 6L1326 from Lingjing. White bracket indicates the area where impact scars are present. Scales = 1 cm. See Tables 2 and 3 for additional information.

Six limb bone fragments and one antler of an axis deer bear evidence for having been used as soft hammers (Table 2). Specimen 6L1326 (Fig 2) is a fragment of a limb bone from a medium size mammal that presents both weathered and fresh fractures. The periosteal surface is slightly eroded and marginally affected by root etching. The fragment features, close to one end, a palimpsest of irregular deep scores, trihedral impacts with rough internal morphology, and removals of cortical lamellae. On the medullar surface, close to the same end, few contiguous flake scars modify the bone lateral morphology.

**Table 2:** Descriptive and morphometric data on Lingjing (layer 11) bone retouchers.

Catalogue no.	Animal size	Species	Element	Lateral Fractures	Distal End Fracture	Proximal End Fracture	Number of knapping areas	Length (mm)	Width (mm)	Thickness (mm)	Cortical thickness (mm)
**6L1326**	M	indet.	Limb Bone	F & W	W	W	1	84.02	24.86	13.38	7.57
**6L1657**	M	Cervid sp.	Metapodial	W	R	W	1	81.29	23.95	16.23	6.94
**6L1881**	M	Cervid sp.	Metapodial	W	W	R	1	63.31	22.33	11.9	7.7
**6L1980**	L	indet.	Limb Bone	F	F	F	2	107.01	48.79	28.41	11.82
**6L2191**	L	indet.	Limb Bone	F	F	F	1	133.95	37.58	20.95	8.93
**7L603**	L	indet.	Limb Bone	F	F	F	1	110.15	42.13	26.96	12.45
**9L0151**	S-M	*Axis shansius*	Antler	—	R	R	1	216.74	26.57*	24.69*	4.67**

Animal size: S = Small; M = Medium; L = Large

Fractures: F = Fresh; R = Recent; W = Weathered

* Calculated to the mid-point of the first beam section

** Calculated at the base

**Fig 3:**
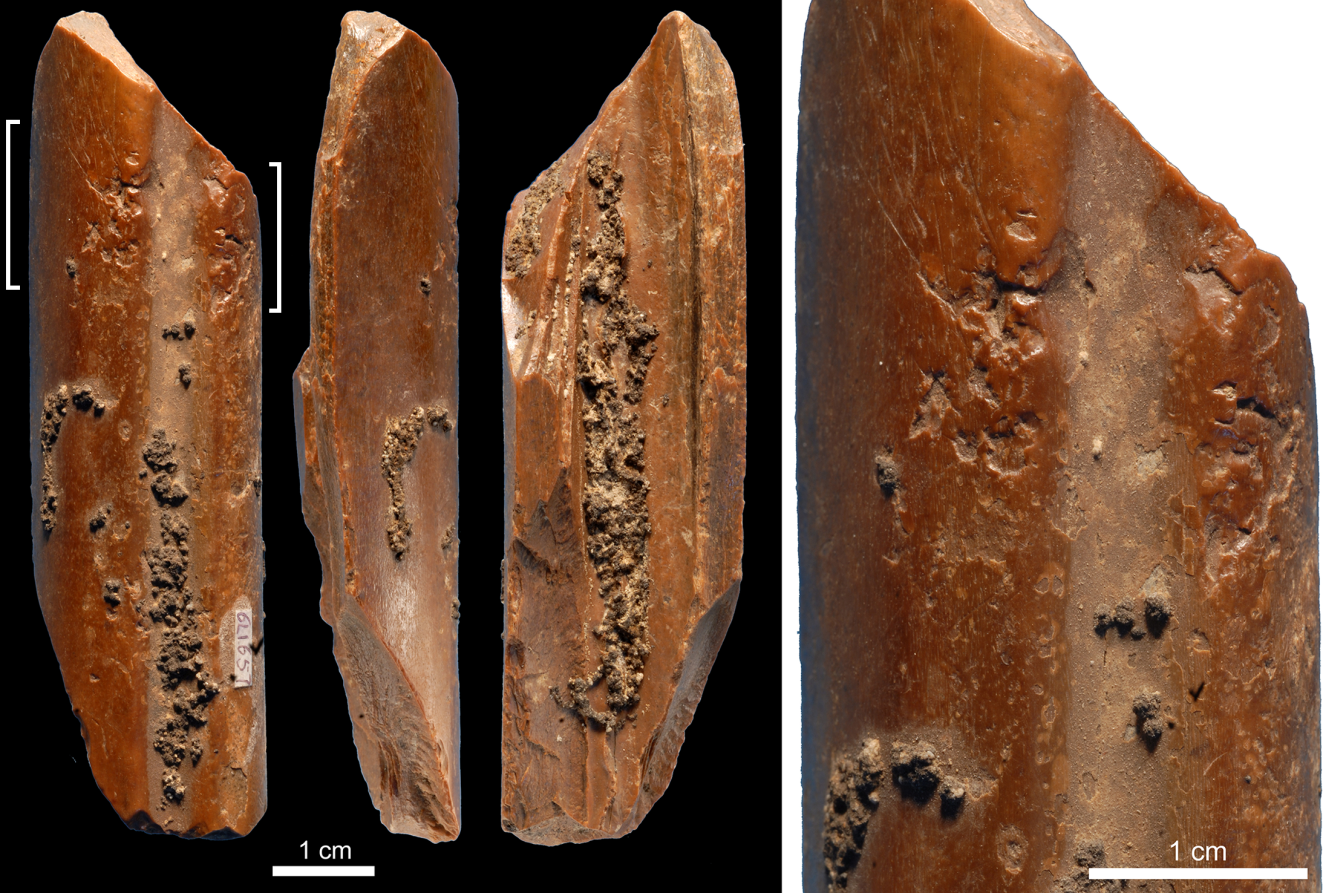
Retoucher 6L1657 from Lingjing. White brackets indicate the areas where impact scars are present. Scales = 1 cm. See Tables 2 and 3 for additional information.

Specimen 6L1657 (Fig 3) is a mesial fragment of a cervid metapodial featuring weathered fractures, cut marks, and exfoliation of primary bone lamellae. Both periosteal and medullar surfaces are partially covered with concretions. At one end, two clusters of impacts with even internal morphology, irregular or trihedral in shape, are located on the ridges flanking the metapodial central trough. These impact scars are associated with scores and removals of primary bone lamellae. Striations originating from the impact scars suggest that, in some instances, the lithic edge kept occasional contact with the bone surface after the blow. Continuous flake scars may have been removed to thin the opposite end of the bone.

**Fig 4:**
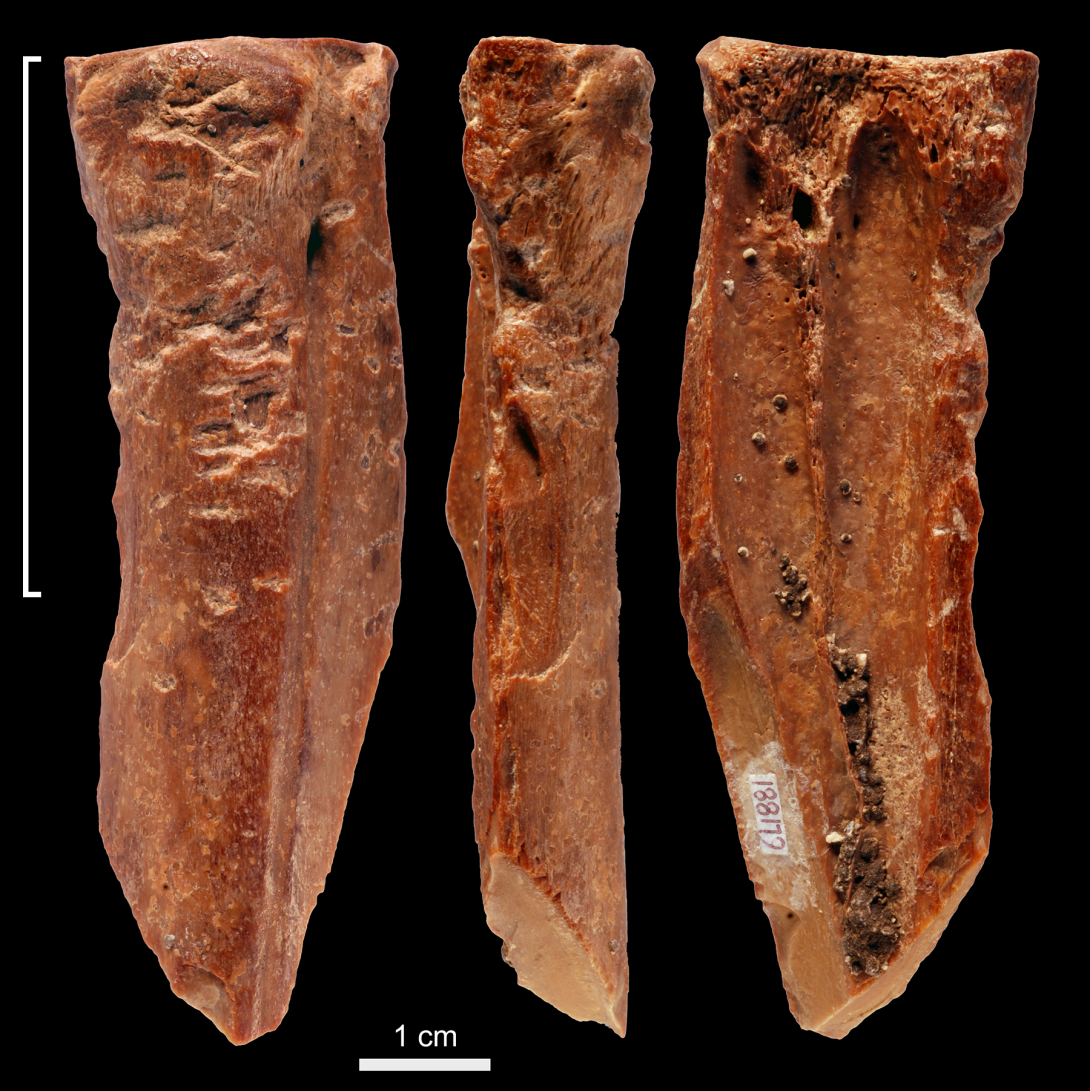
Retoucher 6L1881 from Lingjing. White bracket indicates the area where impact scars are present. Scale = 1 cm. See Tables 2 and 3 for additional information.

Specimen 6L1881 (Fig 4) is a proximal fragment of a cervid metapodial that bears evidence of weathered and recent fractures, and few concretions. A palimpsest of deep sub-rectangular scores, trihedral impact scars, and removals of primary bone lamellae covers half of the periosteal surface. The internal morphology of the stigmata is rough. Few flake scars are observed on one edge of the periosteal surface close to the area bearing scores and pits.

**Fig 5:**
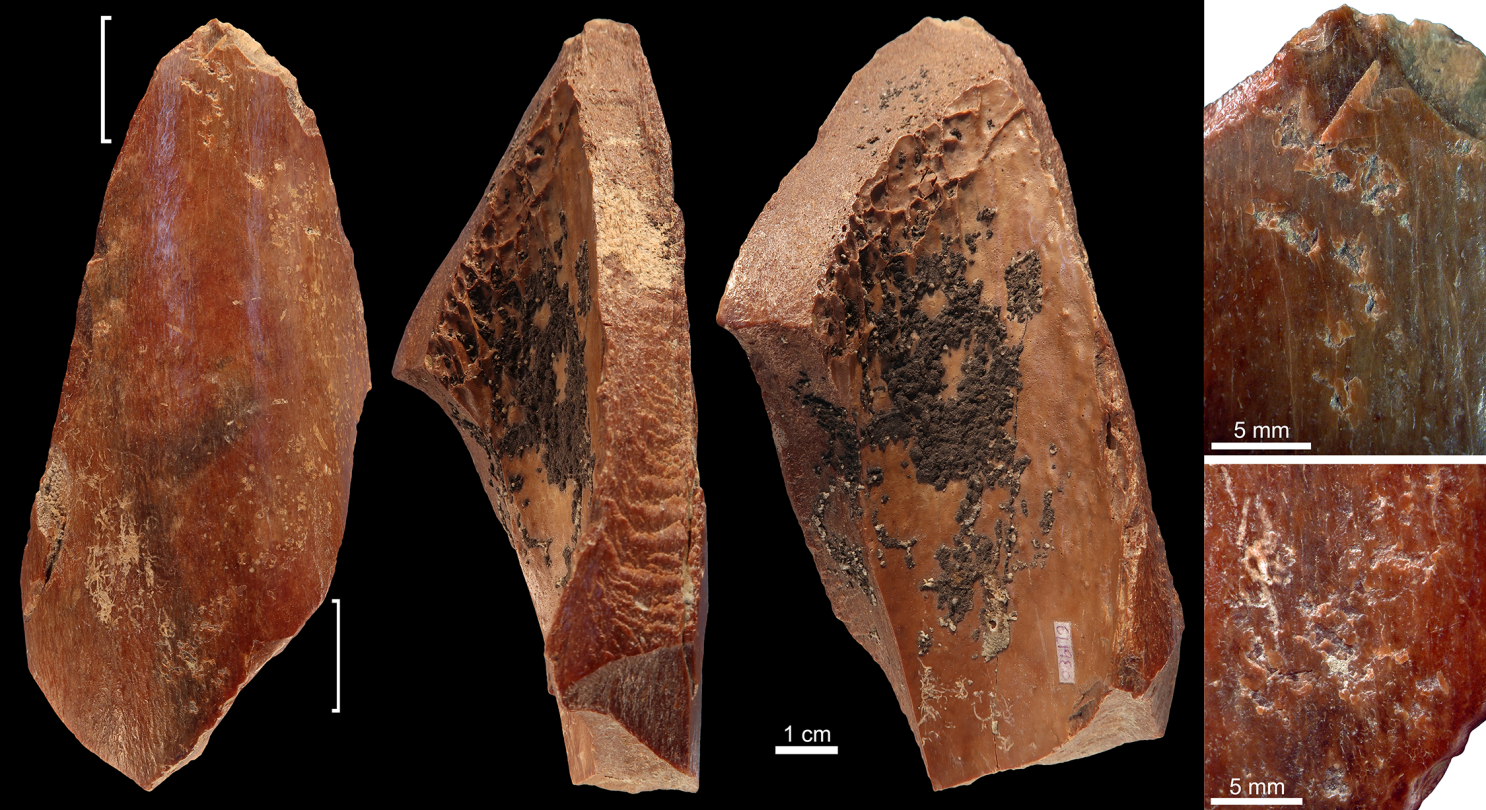
Retoucher 6L1980 from Lingjing. White brackets indicate the areas where impact scars are present. Scale (macro) = 1 cm; scales (micro) = 5 mm). See Tables 2 and 3 for additional information.

Specimen 6L1980 (Fig 5) is a long bone fragment from an undetermined large size mammal showing fresh fractures, cut marks, root etching, and concretions. Both ends present small clusters of scattered trihedral impact scars with rough internal morphology. One edge is modified by continuous marginal flake removals on the medullar and the periosteal surfaces.

**Fig 6:**
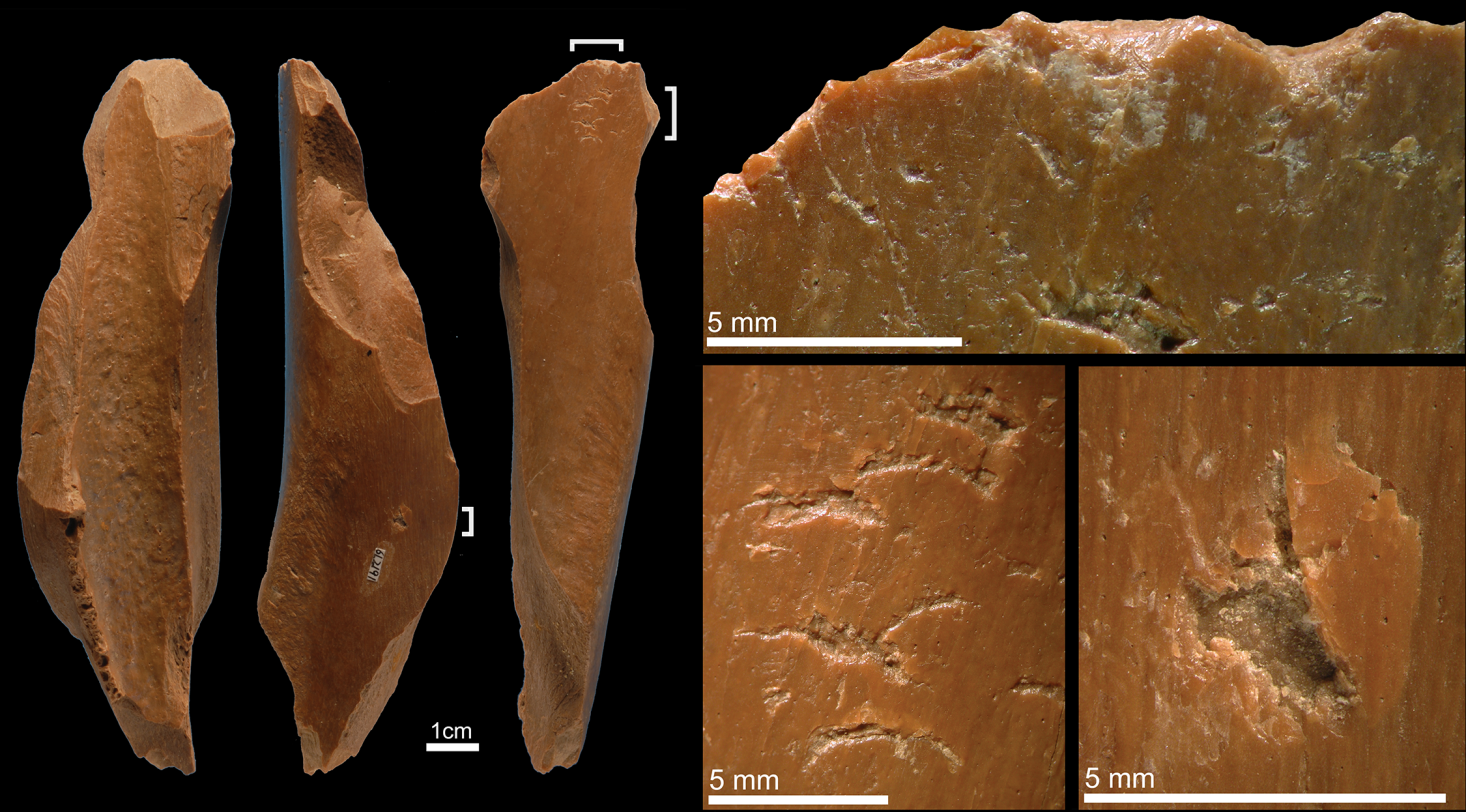
Retoucher 6L2191 from Lingjing. White brackets indicate the areas where impact scars are present. Scale (macro) = 1 cm; scales (micro) = 5 mm). See Tables 2 and 3 for additional information.

Specimen 6L2191 (Fig 6) is a long bone fragment from an undetermined large size mammal that bears evidence of fresh fractures, cut marks, and periosteal flake removals. It features, at one end, a cluster of curved scores with even internal morphology produced while retouching the same cutting edge. Closer to the edge, retouching a sharp lithic point produced microscopic trihedral impacts and flake scars. A single large trihedral impact with a rough internal morphology associated with the uplifting of the primary bone lamellae is observed on another area of the periosteal surface (Fig 6).

**Fig 7:**
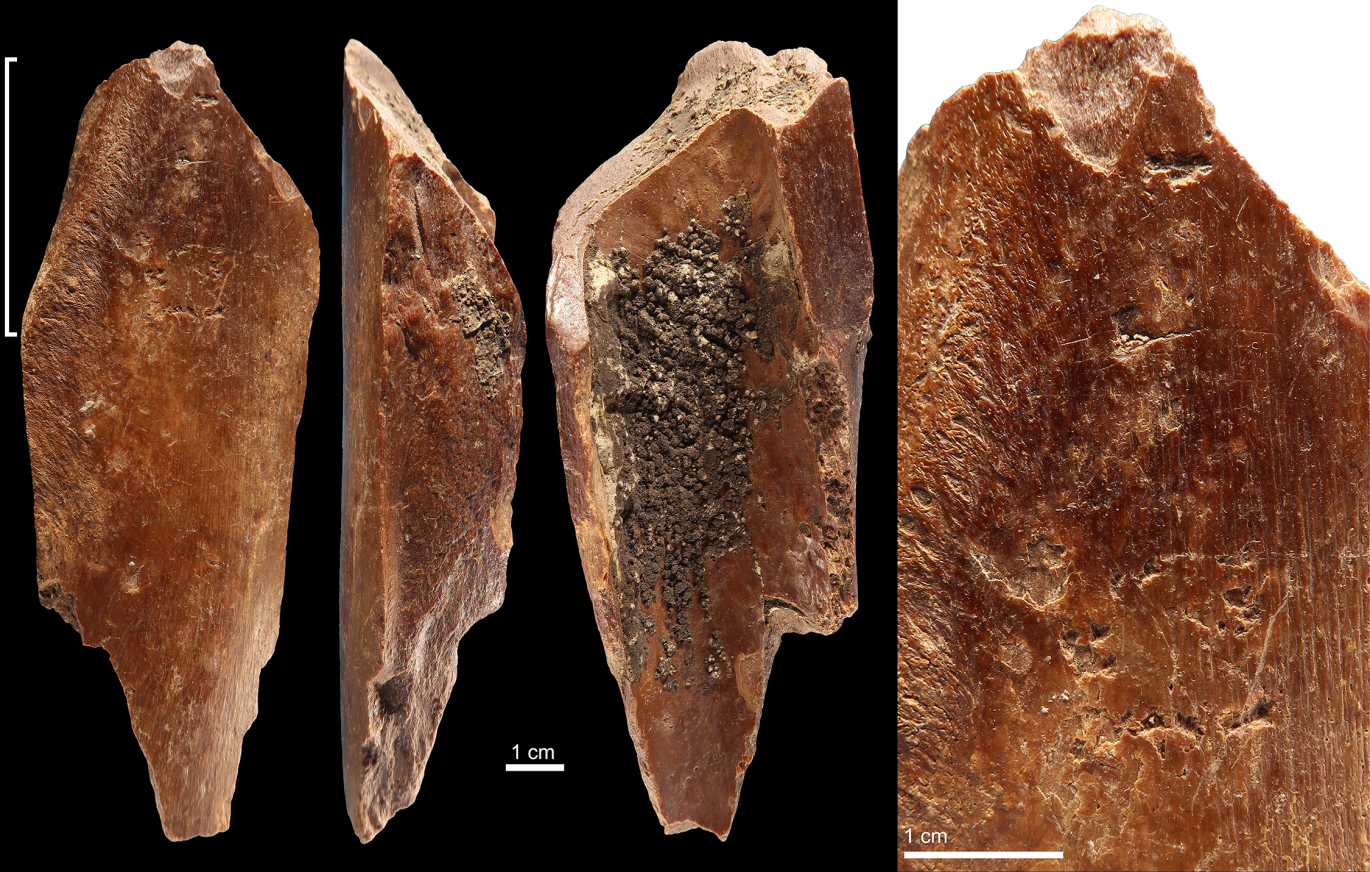
Retoucher 7L603 from Lingjing. White bracket indicates the area where impact scars are present. Scales = 1 cm. See Tables 2 and 3 for additional information.

Specimen 7L603 (Fig 7) is a long bone fragment from an undetermined large size mammal that bears evidence of fresh fractures, cut marks, exfoliation of the primary bone lamellae, and concretions. It features at one end a cluster of scores as well as trihedral and sub-circular impact scars. Both scores and scars display a rough internal morphology and a number are associated with the removal of primary bone lamellae.

**Fig 8:**
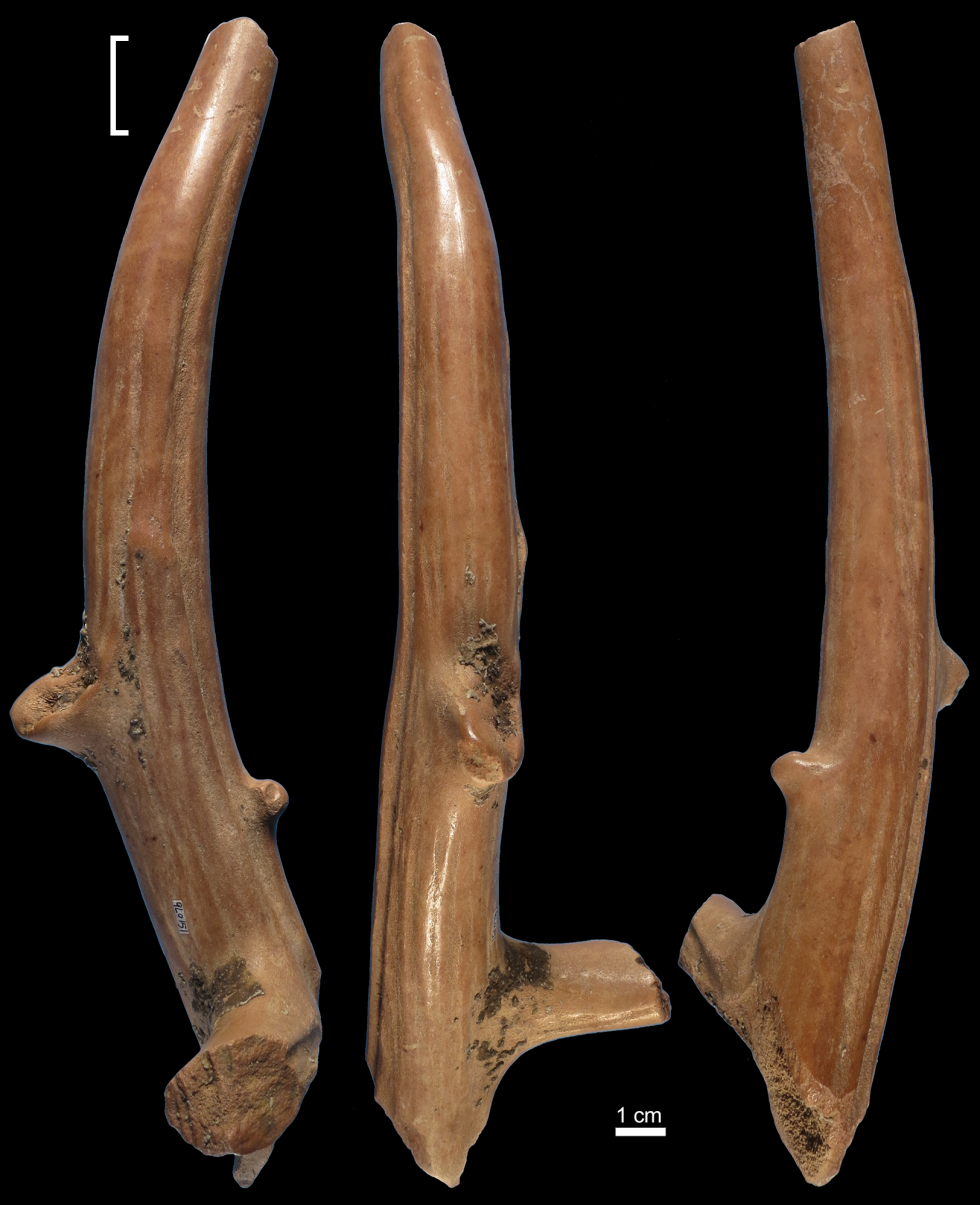
Retoucher 9L0151 from Lingjing. White bracket indicates the area where impact scars are present. Scale = 1 cm. See Tables 2 and 3 for additional information.

**Fig 9:**
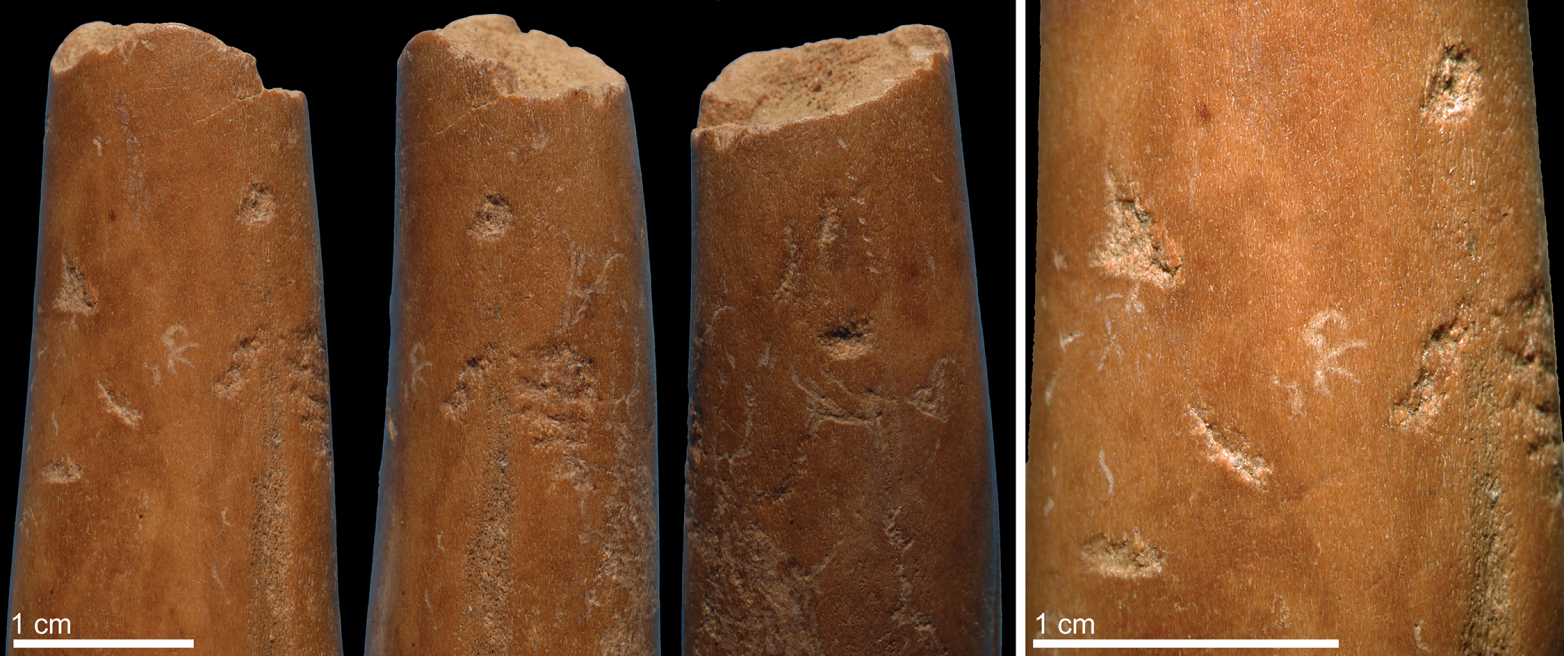
Close up views of the retoucher 9L0151 from Lingjing. Scales = 1 cm. See Tables 2 and 3 for additional information.

Specimen 9L0151 (Fig 8) is an incomplete right antler of an *Axis shansius* subadult. The surface is slightly damaged by root etching. The base, the first tine, and the tip tine present recent fractures. The trez tine [67] shows an old fracture. A few concretions coat the proximal half of the antler, at the junction of the main beam and both the first tine and trez tine. A number of sub-circular and trihedral impact scars as well as linear scores are present on the anterior, lateral and posterior aspects of the antler tip (Fig 9). Most scores cluster in a small area on the lateral aspect. Both scores and scars have a rough internal morphology.

### Summary

**Fig 10:**
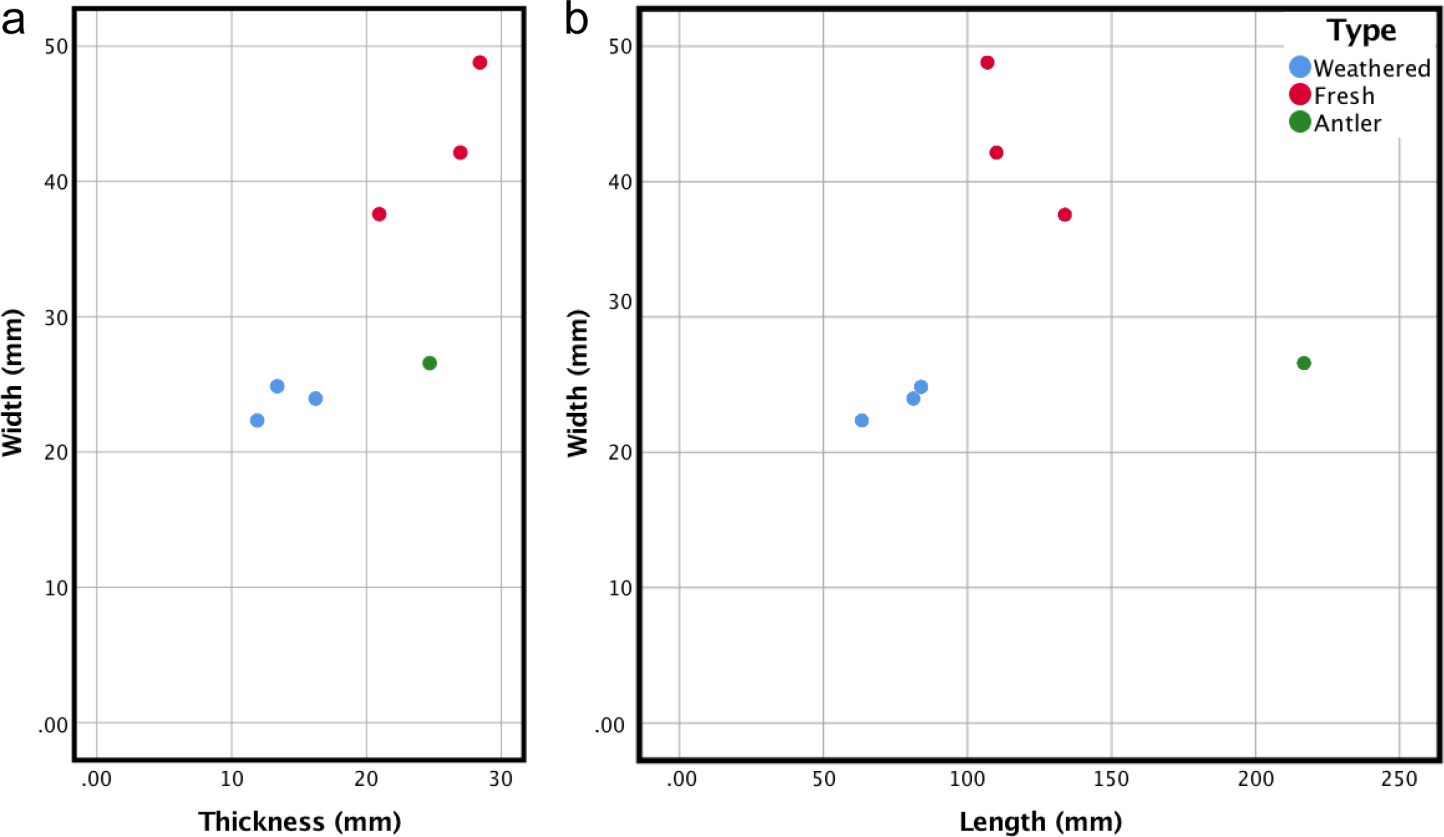
a) Comparison between the width and thickness of Lingjing osseous retouchers; b) Comparison between the width and length of Lingjing osseous retouchers.

Three types of soft hammers were recovered at Lingjing (Tables 2 and 3). The first type consists of weathered limb bone fragments, mostly mesial splinters of cervid metapodials, which were marginally shaped by retouching and intensively utilized on a single area (6L1326, 6L1657, 6L1881). The second type corresponds to limb bone fragments from large size mammal bearing fresh fractures. Traces of their utilization as retouchers occur on one or more areas and generally consist of few shallow impacts left by knapping the same cutting edge during a single session (6L1980, 6L2191, 7L603). These two types differ in size, the former being substantially smaller, and comparatively more elongated and standardized (Fig 10). The third type, represented by a single specimen, consists of a cervid antler bearing, close to its tip, impacts produced by percussing different lithic blanks.

**Table 3:** Technological data on Lingjing (layer 11) bone retoucher and soft hammer.

Catalogue no.	Used area length (mm)	Used area width (mm)	Number of Sub-spherical pits	Number of trihedral pits	Number of scores	Number of scale removals	Lateral Shaping	Internal Morphology
**6L1326**	19.95	8.42	—	20	18	29	Yes	Rough
**6L1657**	13.32	16.58	—	11	6	14	Yes	Even
**6L1881**	40.91	14.62	—	14	23	4	Yes	Rough
**6L1980 Dist.**	12.84	10.95	—	15	—	14	Yes	Rough
**6L1980 Prox.**	10.59	8.92	—	9	—	8	Yes	Rough
**6L2191**	12.05	13.69	—	15	24	1	—	Even and Rough
**7L603**	13.41	11.16	8	18	10	12	—	Rough
**9L0151**	15.75	8.1	4	3	9	—	—	Rough

## Discussion

Archaeozoological, technological, functional, and morphometric results highlight that soft hammers used at Lingjing reflect three distinct behavioural strategies. The first entailed the use of large bone flakes resulting from butchery of large herbivores that were utilized as such for expedient retouching or resharpening of stone tools, likely during carcass processing. These objects were quickly discarded after their utilization. The second involved the fracture of weathered bone from medium size herbivores to obtain elongated splinters and, in some instance, their shaping by percussion to obtain sub-rectangular artefacts. Traces observed on these objects indicate intensive and possibly recurrent utilization, which implies their curation over time. The last corresponds to the occasional use of curated antlers as soft hammer, which may also have fulfilled other functions. The above highlights different logics for the production and use of bone retouchers. The first retoucher type satisfies an immediate need. The second implies long-term planning, and the modification of the blank to impose a shape to the tool, in order to improve its ergonomics, transportability, and efficiency. The third is probably a multi-purpose implement occasionally used as an expedient soft hammer.

These results have implications for the ongoing debate on the nature and distinctiveness of the Chinese Palaeolithic. Most Chinese lithic industries dated between 300 ka and 40 ka are characterized by the persistence of core-and-flake knapping, the use of poor-quality local raw materials, the rarity of prepared cores and their reduction with direct hard hammer percussion, bipolar percussion, and block-on-block technique, the absence of evidence for soft hammer percussion, the rarity of retouched flakes, and the lack of obvious temporal trends [38]. This pattern contrasts with penecontemporaneous technical innovations (raw material selection, Levallois debitage, use of soft hammer, systematic shaping of stone tools by retouch) signalling the emergence of the Middle Palaeolithic in the remainder of Eurasia. The absence of Middle Palaeolithic technologies in large areas of China is considered by some to be the consequence of relatively stable environmental conditions, absence of large-scale population replacement events, low-intensity resources exploitation, high group mobility, the production of perishable tools made of bamboo, and a preference for simple yet flexible stone tool technologies [38,68–73]. As a result, a number of researchers have argued that the term Middle Palaeolithic has no real meaning in most of East Asia [36,37,39,74,75] and should be restricted to assemblages located in peripheral areas, i.e., the Ningxia Autonomous Region [36], the Jilin Province [76,77], and the Inner Mongolia [78], where Middle Palaeolithic diagnostics are present in the lithic assemblages. Others use this term conventionally to designate occupations falling within the range of 300 ka to 40 ka BP, or yielding remains of archaic *Homo sapiens*. Finally, some contend that innovative technologies are present in Chinese assemblages of this period and that their attribution to the Middle Palaeolithic is therefore fully justified [35].

The first obvious implication of our results for the above debate is that soft hammers were used for knapping during the early Late Pleistocene of East Asia, and that this occurred outside the regions in which the Levallois technique is documented. The three types of soft hammers identified at Lingjing demonstrate a good knowledge of the properties of osseous materials for knapping purposes. The shaping and probable curation of one tool type suggest that these tools represented an integral element of the toolkit used by Lingjing hominins. This is also supported by the analysis of the traces of utilization. The overall rough internal morphology of scores and pits indicates, based on experimental results [6], that the three soft hammer types were used to knap coarse lithic raw material such as quartz or quartzite, which represents more than 99% of the knapped lithics found in layer 11. Bone retouchers were therefore essential in knapping activities and it is very likely that additional specimens will be identified in the course of the ongoing reassessment of the Lingjing faunal assemblage. It is also very likely that soft hammers made of bone or antler represent an unrecognized feature of early Late Pleistocene or older Chinese assemblages. The traces of their use bear some similarities with bone modifications produced by other agents and these traces were not systematically searched for during the taphonomic analysis of most Chinese bone assemblages dated to this period.

A broader implication of our results concerns the nature of East Asian early Late Pleistocene cultural adaptations. The behavioural complexity revealed by Lingjing soft hammers suggests that the apparent simplicity of this lithic technology and that of other contemporaneous sites may be illusory as lithics only represented an aspect of past cultural adaptations. The discovery of bone tools effectively complements what we know about these technical systems and highlights behavioural consistencies that could not be inferred from other cultural proxies. These consistencies provide a new dimension to the debate surrounding the existence of the Middle Palaeolithic in this region of the world. The attribution of East Asian sites to the Middle Palaeolithic is generally based either on their chronology or on the occurrence of cultural traits pertaining to lithic technology such as the Levallois debitage. This approach assumes that the chosen traits represent evolutionary hallmarks applicable to regions of the world different from those in which they were originally identified. An alternative approach, that we wish to promote here, consists in identifying original characters of regional cultural trajectories from a variety of aspects of past material culture, and understanding the behavioural and cognitive implications they may have had for past hominin populations.
